# Homozygous mutations in *VAMP* *1* cause a presynaptic congenital myasthenic syndrome

**DOI:** 10.1002/ana.24905

**Published:** 2017-03-29

**Authors:** Vincenzo Salpietro, Weichun Lin, Andrea Delle Vedove, Markus Storbeck, Yun Liu, Stephanie Efthymiou, Andreea Manole, Sarah Wiethoff, Qiaohong Ye, Anand Saggar, Kenneth McElreavey, Shyam S. Krishnakumar, SYNAPS Study Group, Matthew Pitt, Oscar D. Bello, James E. Rothman, Lina Basel‐Vanagaite, Monika Weisz Hubshman, Sharon Aharoni, Adnan Y. Manzur, Brunhilde Wirth, Henry Houlden

**Affiliations:** ^1^ Department of Molecular Neuroscience, Institute of NeurologyUniversity College London Institute of NeurologyLondonUnited Kingdom; ^2^ Department of NeuroscienceUniversity of Texas Southwestern Medical CenterDallasTX; ^3^ Institute of Human Genetics, Center for Molecular Medicine CologneCologneGermany; ^4^ Institute for GeneticsUniversity of CologneCologneGermany; ^5^ St George's Hospital, National Health Service Foundation TrustLondonUnited Kingdom; ^6^ Human Developmental Genetics, Pasteur InstituteParisFrance; ^7^ Department of Cell BiologyYale School of MedicineNew HavenCT; ^8^ Department of Clinical and Experimental EpilepsyUniversity College London Institute of NeurologyLondonUnited Kingdom; ^9^ Department of Clinical NeurophysiologyGreat Ormond Street Hospital for Children, National Health Service Foundation TrustLondonUnited Kingdom; ^10^ Pediatric Genetics Unit, Schneider Children's Medical Center of IsraelPetach TikvaIsrael; ^11^ Raphael Recanati Genetic Institute, Rabin Medical CenterPetach TikvaIsrael; ^12^ Sackler Faculty of MedicineTel Aviv UniversityTel AvivIsrael; ^13^ Institute of Child Neurology, Schneider Children's Medical Center of IsraelPetach TikvaIsrael; ^14^ Department of Pediatric NeurologyDubowitz Neuromuscular Centre, Great Ormond Street Hospital for Children National Health Service Foundation TrustLondonUnited Kingdom

## Abstract

We report 2 families with undiagnosed recessive presynaptic congenital myasthenic syndrome (CMS). Whole exome or genome sequencing identified segregating homozygous variants in *VAMP1*: c.51_64delAGGTGGGGGTCCCC in a Kuwaiti family and c.146G>C in an Israeli family. *VAMP1* is crucial for vesicle fusion at presynaptic neuromuscular junction (NMJ). Electrodiagnostic examination showed severely low compound muscle action potentials and presynaptic impairment. We assessed the effect of the nonsense mutation on mRNA levels and evaluated the NMJ transmission in *VAMP1* ^*lew/lew*^ mice, observing neurophysiological features of presynaptic impairment, similar to the patients. Taken together, our findings highlight *VAMP1* homozygous mutations as a cause of presynaptic CMS. Ann Neurol 2017;81:597–603

The congenital myasthenic syndromes (CMSs) are a heterogeneous group of inherited diseases of the neuromuscular junction (NMJ), with fatigable muscle weakness as the clinical hallmark.^1^ Several molecular causes can be implicated in CMS pathophysiology, including mutations in genes encoding proteins associated with the muscle nicotinic acetylcholine receptor and the synaptic basal lamina, or (more rarely) involved in the NMJ presynaptic transmission.^2^, ^3^, ^4^, ^5^, ^6^

We describe 2 families from Kuwait and Israel where 2 of the siblings in each family presented clinical and neurophysiological features typical of a presynaptic CMS. Whole exome sequencing (WES) or whole genome sequencing (WGS) followed by Sanger sequencing unraveled either a homozygous frameshift or missense variants in *VAMP1* segregating with the phenotype in the 2 families. Screening a cohort of 63 undiagnosed CMS individuals failed to show any further causative variant in *VAMP1*.

## Materials and Methods

### Subjects

This study was approved by the institutional review boards of the participating centers. Informed consent was obtained from the families. Clinical details were obtained from medical records. Neurophysiological studies were performed according to standard procedures.^7^, ^8^

### Genetic Studies

Before WES, the Kuwaiti probands (Family 1) underwent extensive molecular investigations that included sequencing of *AGRN*, mitochondrial DNA (mtDNA) sequencing, and deletion/duplication analysis and array comparative genome hybridization, which were all negative. Clinical trio‐based WES of Family 1 and WGS of the Israeli probands and their parents (Family 2) were performed as previously described.^9^, ^10^ Immortalized lymphoblastoid cell lines were used for RNA extraction, reverse transcription polymerase chain reaction (RT‐PCR) analysis, and semiquantitative RT‐PCR assay. Sanger sequencing was performed to analyze segregation of the variants identified by WES/WGS.

### *Vamp1* ^*lew/lew*^ Mice

Breeder pairs of *Vamp1* ^*+/lew*^ mice (C3H/HeDiSnJ‐*Vamp1* ^lew^/GrsrJ, stock # 004626) were obtained from the Jackson Laboratory (Bar Harbor, ME) and mated to generate homozygous mutant (*Vamp1* ^*lew/lew*^) mice. Electrophysiological and morphological analyses of the NMJ in the *Vamp1* ^*lew/lew*^ mice were performed as previously reported.^11^, ^12^ All experimental protocols were approved by the University of Texas Southwestern Medical Center institutional animal care and use committee.

## Results

### Clinical and Neurophysiological Characteristics

#### Family 1

**Figure 1:**
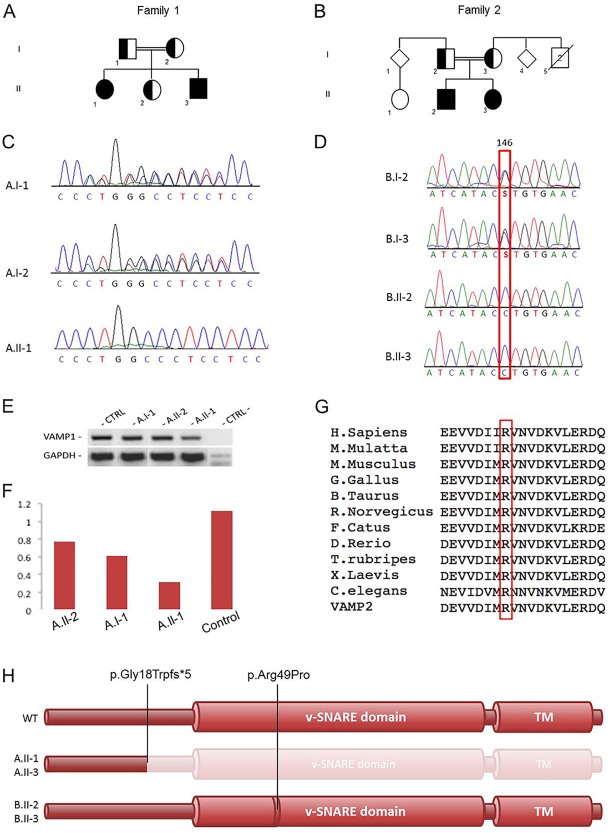
Family trees, Sanger sequencing, and *VAMP1* mutation analysis. (A) Pedigree from Family 1. (B) Pedigree from Family 2. (C) Electropherograms of carrier parents and index case with the c.51_64delAGGTGGGGGTCCCC variant. (D) Electropherograms of carrier parents and the 2 patients with the c.146G>C variant. (E) Reverse transcription polymerase chain reaction (PCR) amplifying the mutant cDNA transcript from mRNA extracted from the immortalized lymphoblastoid cell lines of the index case, her father, and her healthy sister (both carriers of the heterozygous deletion), and a wild‐type control (CTRL). (F) Analysis of the semiquantitative PCR using the densitometry software ImageJ after normalization relative to a housekeeping gene (*GAPDH*) and calculation using a relative relationship method. (G) Multiple‐sequence alignment showing complete conservation of protein sequence across species and SNARE homolog *VAMP2* in the v‐SNARE coiled coil homology, in which the disease‐segregating mutation p.Arg49Pro was found. (H) *VAMP1* protein representative. The c.51_64delAGGTGGGGGTCCCC deletion causes a nonsense mutation, putatively producing a truncated protein lacking the v‐SNARE and the transmembrane (TM) domains, whereas the p.Arg49Pro mutation affects an active site of the conserved v‐SNARE domain.

Both affected individuals A.II‐1 and A.II‐3 (Fig 1A) presented shortly after birth with hypotonia and muscular weakness. Feeding difficulties requiring gavage feeding, delayed motor development, and ophthalmoparesis characterized the disease course. A.II‐3 also presented joint contractures. Creatine kinase and plasma lactate were normal in the 2 children. On initial evaluation of Patient A.II‐3, muscle biopsy showed myopathic features and borderline low complex IV activity (0.011; normal range = 0.014–0.034), but congenital myopathy gene panel and mtDNA analysis were negative. Although hypotonia slightly improved in Patient A.II‐1, at the age of 3 years she still had difficulties standing upright and was unable to walk without support. Electrodiagnostic examination (EDX) in the 2 individuals showed similar findings (Table), with marked reduction in the amplitude of the compound muscle action potentials (CMAPs) and an increase in the amplitude to >200% of baseline on repetitive nerve stimulation (RNS) to 20Hz, indicating presynaptic impairment of NMJ transmission. The children's weakness slightly ameliorated under pyridostigmine treatment.

**Table 1:** Clinical and Neurophysiological Features of *VAMP1*‐Associated Congenital Myasthenic Syndrome in Our Families

Feature	A.II‐1	A.II‐3	B.II‐1	B.II‐2
Parental consanguinity	+	+	+	+
Onset	Birth	Birth	Antenatal, DFM	Birth
Muscle weakness	++	++	++	++
Developmental delay	++	++	++	++
Feeding difficulties	++	++	++	++
Ophthalmological abnormalities	Strabismus, mild ophthalmoplegia	Mild ophthalmoplegia	Strabismus	Strabismus
GI abnormalities	−	GERD	Dysphagia	Dysphagia
Skeletal joint abnormalities	−	Joint contractures	Joint laxity, kyphoscoliosis	Joint contractures
Chest infections, aspiration	+	+	+	+
Response to pyridostigmine	+	+	+	+
Sensory studies	Normal	Normal	Normal	NT
Motor studies	AH CMAP ↓↓	AH CMAP ↓↓	ACL CMAP ↓↓	NT
EMG	Myopathic	Myopathic	Myopathic	NT
Repetitive stimulation	AH: 3Hz, + 32.8%; 20Hz, + 640%	AH: 3Hz, + 60%; 20Hz, + 207%	NA	NT
Jitter	EDC, no twitch	Orb oculi, no twitch	↑↑ mean MCD = 74.3 µs	NT

ACL = accessorius motor left; AH = abductor pollicis; CMAP = compound muscle action potential; DFM = decreased fetal movements; EDC = extensor digitorum communis; EMG = electromyogram; GERD = gastroesophageal reflux disease; GI = gastrointestinal; MCD = mean consecutive difference; NA = not available; NT = not tested; Orb oculi = orbicularis oculi.

#### Family 2

The affected individuals of this family (B.II‐2 and B.II‐3; see Fig 1B) showed severe hypotonia and muscle weakness since birth. Both siblings had feeding difficulties and required percutaneous endoscopic gastrostomy. They presented severe impairment of developmental milestones. B.II‐1 also showed joint laxity and kyphoscoliosis. B.II‐3 presented knee contractures and breathing difficulties. During disease course, both children showed markedly reduced ability to generate antigravity posture and movements. B.II‐2 never reached autonomous walk; his EDX showed severely low CMAPs and increased neuromuscular jitter, indicating NMJ transmission abnormalities (see Table 1). In both siblings, pyridostigmine treatment improved symptoms.

### Identification of the *VAMP1* Mutation

Trio‐based WES of Family 1 (A.I‐1, A.I‐2, A.II‐1; see Fig 1A) indicated in the index case 3 genes (Supplementary Table 1) carrying homozygous exonic variants predicted to have a possible pathogenic effect on protein function, based on the guidelines for variant classification.^13^ Full Sanger‐based segregation analysis of the candidate variants reduced the gene list to only 1 mutation in *VAMP1* (NM_014231: c.51_64delAGGTGGGGGTCCCC; p.Gly18TrpfsTer5*), which was found to be homozygous in the affected individuals and heterozygous in their healthy sister and in the unaffected parents (see Fig 1C; data shown for the index case and her parents).

WGS of the 4 members of Family 2 (B.I‐2, B.I‐3, B.II‐2, B.II‐3; see Fig 1B) identified 6 genes carrying rare (likely) damaging variants (Supplementary Table 2), which were homozygous in the affected individuals and heterozygous in the parents.^12^ Among these 6 variants, a homozygous missense mutation in *VAMP1* (NM_014231: c.146G>C; p.Arg49Pro; see Fig 1D) emerged as the most likely explanation for the disease pathogenesis, as supported by protein function (the mutation affects a conserved amino acid within the active domain of the protein),^14^, ^15^ expression and role of this gene in the NMJ,^12^, ^16^ and the homozygous mutation identified in the patients from Family 1 presenting the same phenotype (see Fig 1C–G).

RT‐PCR assay (performed to analyze possible nonsense‐mediated decay associated with the *VAMP1* truncating variant in Family 1) found a mild reduction of mutant cDNA expression in the index case compared to the heterozygous carriers and the wild‐type control (see Fig 1E, F).

### Impairments of the Neuromuscular Junction in *Vamp1* ^*lew/lew*^ Mice

**Figure 2:**
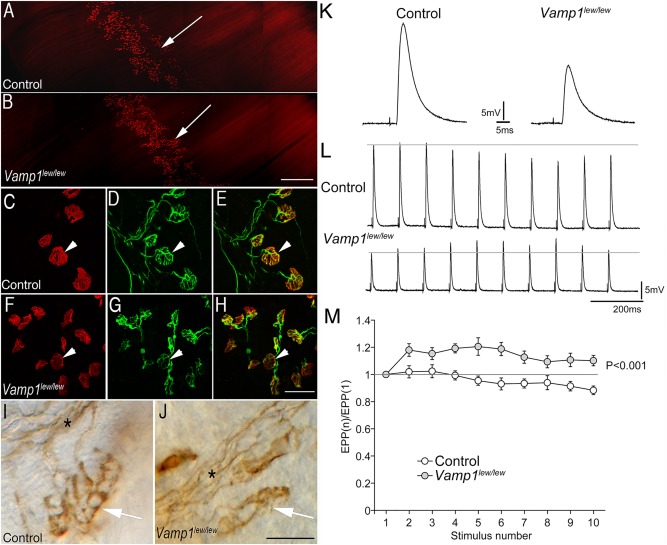
Synaptic defects at the neuromuscular junctions in *Vamp1* ^*lew/lew*^ mice. (A, B) Low‐power images of the whole‐mount diaphragm muscles (P14) labeled by Texas Red—conjugated α‐bungarotoxin. The endplate band *(arrow)* is similarly localized along the central regions of the muscle in both control (A) and *Vamp1* ^*lew/lew*^ mice (B). (C–H) High‐power confocal images of individual neuromuscular synapses in triangularis sterni muscles, labeled by Texas Red–conjugated α‐bungarotoxin (*arrowheads* in C and F) and antineurofilament NF150 and antisynaptotagmin2 antibodies (*arrowheads* in D and G point to the nerve terminals). Merged images are shown in E and H, for control and *Vamp1* ^*lew/lew*^ mice, respectively. (I, J) Individual neuromuscular synapses *(arrows)* in triangularis sterni muscles labeled by antisyntaxin1 antibodies. The synapses are markedly smaller in *Vamp1* ^*lew/lew*^ mice compared with the control. Asterisks indicate nerve bundles. (K) An example of endplate potentials (EPPs) recorded in the diaphragm muscle in control and *Vamp1* ^*lew/lew*^ mice. (L) EPP traces responding to a low‐frequency, repetitive nerve stimulation (10Hz). (M) Quantitative measurement of the ratios of EPP amplitudes: EPP(n) to the first EPP amplitude, (EPP1). A low‐frequency, repetitive stimulation (10Hz) led to a run‐down of EPPs in control, but synaptic facilitation in (*Vamp1* ^*lew/lew*^) mice.

To further investigate whether a biallelic null mutation in *VAMP1* in animal models may cause presynaptic NMJ abnormalities similarly to affected individuals, we re‐examined *Vamp1* ^*lew/lew*^ mutant mice that were previously described.^11^, ^12^ The endplates were localized along the central regions of the muscle in both control and *Vamp1* ^*lew/lew*^ mice (Fig 2). Individual neuromuscular synapses were found markedly smaller in *Vamp1* ^*lew/lew*^ mice compared with control, and a severe reduction in endplate potentials (EPPs) was also observed in the mutant mice. Importantly, a low‐frequency, repetitive stimulation (10Hz) led to a run‐down of EPPs in control mice, but synaptic facilitation in *Vamp1* ^*lew/lew*^ mice, indicating presynaptic defects.

## Discussion

Here, we report 4 children from 2 consanguineous families who presented with typical clinical and neurophysiological features of presynaptic CMS associated with homozygous mutations in *VAMP1*.

The protein encoded by this gene is a member of the synaptobrevin family.^14^ Synaptobrevins (eg, Vamp1, Vamp2), syntaxins, and the synaptosomal‐associated protein Snap25 represent the main components of the SNARE (soluble N ‐ethylmaleimide‐sensitive factor attachment protein receptors) complex, which is involved in docking and fusion of synaptic vesicles with the presynaptic membrane at the central and the neuromuscular synapses.^15^, ^16^ Proteins belonging to this complex are involved in vesicle docking through the evolutionarily conserved active v‐SNARE coiled coil homology domain and present high sequence similarity across the different SNAREs.^17^, ^18^, ^19^

Notably, the c.51_64delAGGTGGGGGTCCCC frameshift deletion identified in Family 1 leads to a change in the gene reading frame with the generation of a premature stop codon 5 amino acids downstream (see Fig 1H). The result is a putative *VAMP1* product of only 21 amino acids, with a resulting function that is highly likely to be disrupted due to the absence of the downstream v‐SNARE domain (amino acids 33–93). The homozygous mutation identified in Family 2 consists of a substitution of a highly conserved arginine (Gerp++ score = 5.77) by a proline within the v‐SNARE domain. The mutated arginine residue in position 49 corresponds to the arginine in position 47 of the better‐studied SNARE homolog *VAMP2*, encoding another synaptobrevin with similar functions to *VAMP1*.^20^

Interestingly, it has been shown that disruption of this specific site in *VAMP2* interferes with SNARE complex assembly, impairing neurotransmission, likely due to lack of association with other proteins involved in vesicle fusion.^21^, ^22^, ^23^ The Arg49Pro mutation is predicted as deleterious by SIFT, PolyPhen, and Mutation Taster and is carried in the heterozygous state by only 1 individual in the ExAC database (http://exac.broadinstitute.org, last accessed January 2017). Of note, in the ExAC database of 60,706 individuals there are only 17 individuals with heterozygous nonsynonymous single nucleotide substitutions within the v‐SNARE domain and 4 individuals in total carrying heterozygous truncating variants in *VAMP1*. None of these variants is present as homozygous, providing supportive evidence of pathogenicity for biallelic *VAMP1* variants, either resulting in changes of the gene reading frame or affecting conserved active sites crucial to v‐SNARE domain function.

Interestingly, we also showed that the electrodiagnostic anomalies recorded in *VAMP1*‐associated CMS are consistent with the abnormal features of presynaptic transmission we recorded in the *VAMP1* null mutant mice (including the incremental response to RNS; see Fig 2). These animals, of a model called *lethal wasting* (carrying a homozygous mutation that causes the truncation of half of the protein), lack movement because of an impaired NMJ transmission and die within 3 weeks of birth.^11^, ^12^

To date, biallelic variants in *VAMP1* have never been reported, but heterozygous mutations in this gene have been described by a single study in association with a phenotype of autosomal dominant spastic ataxia.^23^ However, we have not observed any neurological phenotype in the heterozygous carriers from the 2 families.

In conclusion, the identification of biallelic variants in *VAMP1* as a novel cause of CMS, in addition to other genes (eg, *SNAP25B, SYT2*) previously associated with similar presynaptic abnormalities of neuromuscular transmission,^5^, ^6^ highlights the crucial role of different SNAREs in NMJ physiology. Intriguingly, the relatively mild phenotype showed by our patients compared to the mouse model, which dies prematurely, suggests the possible existence of species‐specific compensation of vesicle fusion and release at the nerve terminal, perhaps through genetic modifiers in humans but not in mice or fruit flies. This highlights a promising area of future research aimed at the pathways involved in physiological presynaptic vesicle exocytosis at the motor endplate.

## Author Contributions

Study concept and design: W.L., J.E.R., B.W., H.H. Data acquisition and analysis: A.M., A.S., K.M., L.B.‐V., M.W.H., S.A., M.P., S.E., S.W., A.Y.M., M.S., O.B., S.S.K. Drafting the manuscript and figures: V.S., A.D.V., Y.L., Q.Y.

## Potential Conflicts of Interest

Nothing to report.

## Supporting information

Additional supporting information can be found in the online version of this article.

Supporting Information 1
