# Supplementary material for: Homozygous mutations in VAMP 1 cause a presynaptic congenital myasthenic syndrome
Source: Ann Neurol. 2017 Mar 29;81(4):597–603. doi: 10.1002/ana.24905 (PMC5413866; doi:10.1002/ana.24905)
Supplement: Supplementary file 1 — Supporting Information 1 [file ANA-81-597-s001.docx]

**Supplementary Table 1**

Homozygous variants identified in the clinical exome of the index case from Family 1 (A.II-1) present in heterozygous state in both the parents (A.I-1, 1.I-2)

| **Protein**  **Mutation** | **UniProt Function Summary** | **Allele frequency (ExAC)** |
| --- | --- | --- |
| *AP1B1*  c.2245_2248delGCCG  p.Thr750Ala* | Subunit of clathrin-associated adaptor protein complex 1 that plays a role in protein sorting in the late-Golgi/trans-Golgi network (TGN) and/or endosomes. The AP complexes mediate both the recruitment of clathrin to membranes and the recognition of sorting signals within the cytosolic tails of transmembrane cargo molecules | 0  (0 hom) |
| *CACTIN*  c.1610_1615dupGCGAGG  p.Gly537_Glu538dup* | Involved in the regulation of innate immune response. Acts as negative regulator of Toll-like receptor and interferon-regulatory factor (IRF) signaling pathways. Contributes to the regulation of transcriptional activation of NF-kappa-B target genes in response to endogenous proinflammatory stimuli. May play a role during early embryonic development. Probably involved in pre-mRNA splicing | 0  (0 hom) |
| *VAMP1*  c.51_64delAGGTGGGGGTCCCC  p.Gly18TrpfsTer5* | Involved in the targeting and/or fusion of transport vesicles to their target membrane | 0  (0 hom) |

**Supplementary Table 2**

Homozygous variants of possible clinical significance identified in the patients from Family 2 (B.II-2, B.II-3) present in heterozygous state in both the parents (B.I-2, B.I-3)

| **Protein**  **Mutation** | **UniProt Function Summary** | **Allele frequency (ExAC)** |
| --- | --- | --- |
| *APOBEC1*  c.1A>G  p.Met1Val | Catalytic component of the apolipoprotein B mRNA editing enzyme complex which is responsible for the post-transcriptional editing of a CAA codon to a UAA codon for stop in the APOB mRNA. Also involved in CGA (Arg) to UGA (Stop) editing in the NF1 mRNA. May also play a role in the epigenetic regulation of gene expression by participating in DNA demethylation. | 0.00154  (0 hom) |
| *TLR6*  c.2124G>C  p.Gln708His | Receptor for lipopeptides and other molecules, activating the innate immune response. Upon interaction with TLR4, promotes sterile inflammation in response to oxLDL or amyloid-beta 42. | 0.0002965  (0 hom) |
| *RHNO1*  c.46C>G  p.Leu16Val | Plays a role in DNA damage response signaling upon genotoxic stresses during the S phase. Required for the progression of the G1 to S phase transition. Plays a role in the stimulation of CHEK1 phosphorylation. | 0.003682  (1 hom) |
| *PH3H*  c.1904C>T  p.Thr635Met | Catalyzes the formation of 3-hydroxyproline in -Xaa-Pro-Gly-sequences in collagens, especially types IV and V. | 0.01023  (5 hom) |
| *GRXCR1*  c.272G>T | May play a role in actin filament architecture in developing stereocilia of sensory cells. | 0.005064  (7 hom) |
| *VAMP1*  c.146G>C  p.Arg49Pro | Involved in the targeting and/or fusion of transport vesicles to their target membrane | 0.000008238  (0 hom) |
